# Supplementary material for: Tracing Acid-Base Variables in Exercising Horses: Effects of Pre-Loading Oral Electrolytes
Source: Animals (Basel). 2022 Dec 24;13(1):73. doi: 10.3390/ani13010073 (PMC9817799; doi:10.3390/ani13010073)
Supplement: Supplementary file 1 [file animals-13-00073-s001.zip › animals-2010573-supplementary.pdf]

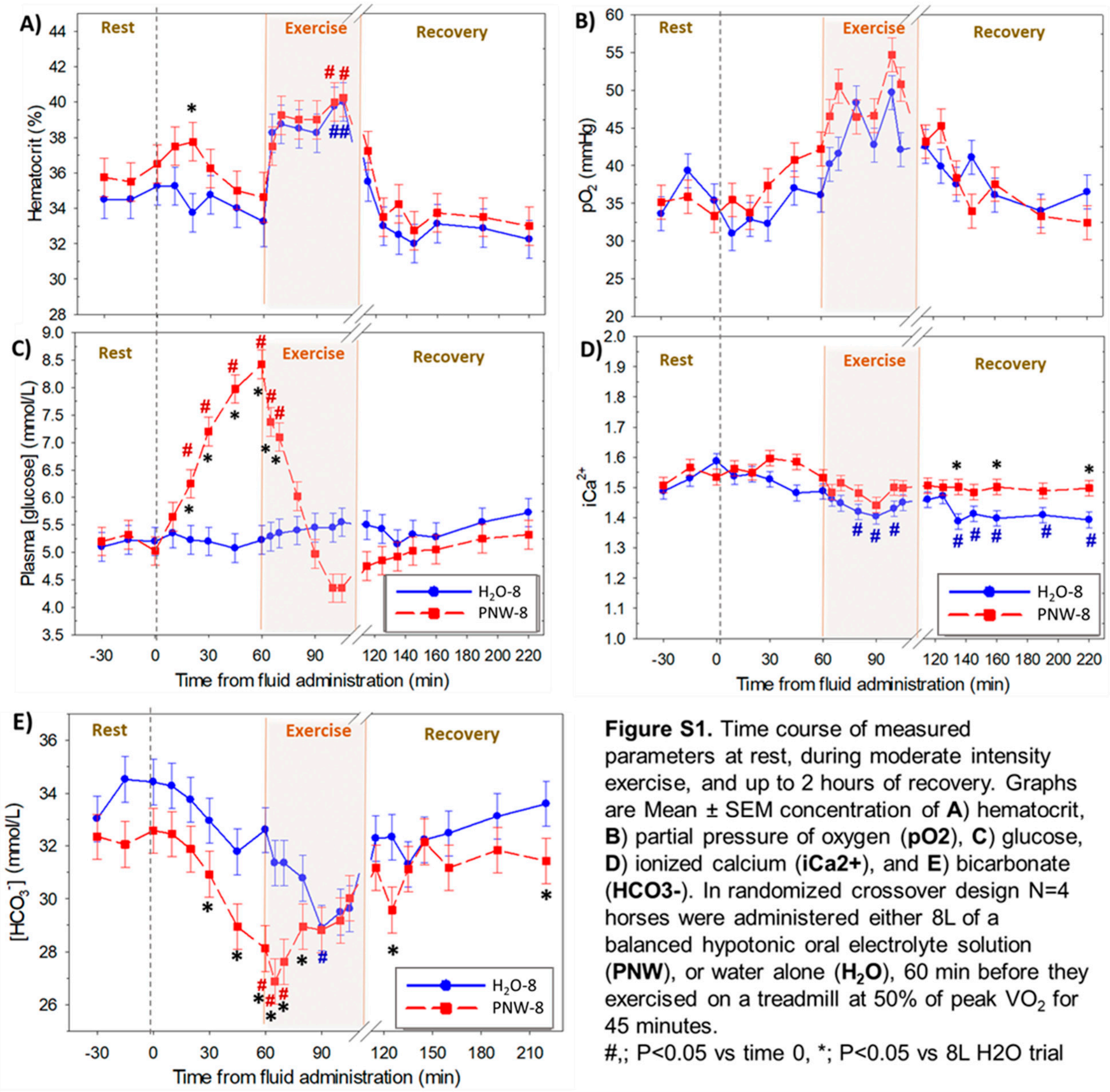

Figure S1. Time course of measured parameters at rest, during moderate intensity exercise, and up to 2 hours of recovery. Graphs are Mean ± SEM concentration of **A**) hematocrit, **B**) partial pressure of oxygen (**pO2**), **C**) glucose, **D**) ionized calcium (**iCa2+**), and **E**) bicarbonate (**HCO3-**). In randomized crossover design N=4 horses were administered either 8L of a balanced hypotonic oral electrolyte solution (**PNW**), or water alone (**H_2O**), 60 min before they exercised on a treadmill at 50% of peak VO_2 for 45 minutes.
#,; P<0.05 vs time 0, *; P<0.05 vs 8L H2O trial

Figure S1. The time courses of supplementary measured parameters at rest, during moderate intensity exercise, and up to 2 hours of recovery. **A**) hematocrit, **B**) partial pressure of oxygen (pO2), **C**) glucose, **D**) ionized calcium ([iCa^{2+}]), and **E**) bicarbonate ([HCO_3^-]). In randomized crossover design n=4 horses were administered either 8L of a balanced hypotonic oral

electrolyte solution (**PNW-8**), or water alone (**H_2O-8**), 60 min before they exercised on a treadmill at 50% of peak VO_2 for 45 minutes. Values are mean ± SE. #,; P<0.05 vs time 0, *; P<0.05 vs H2O-8 trial.

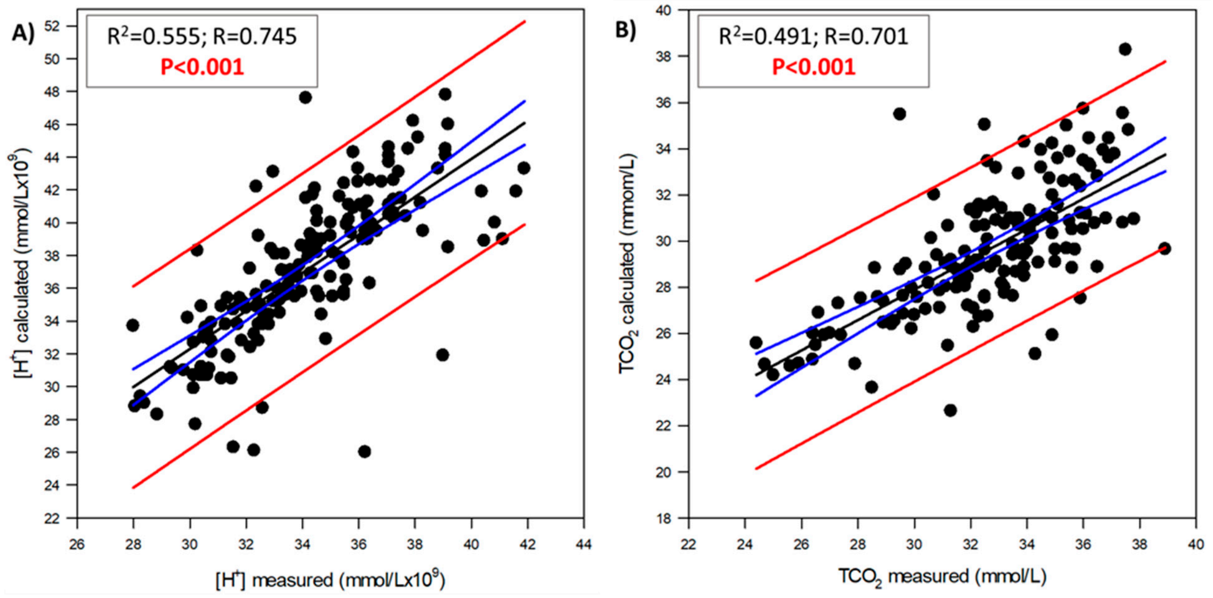

Figure S2. Linear regression analysis showing excellent correlation between the measured and calculated concentrations of independent acid-base variables **A**) hydrogen ion ([H^+]) and **B**) total carbon dioxide (**TCO2**)**.** In randomized crossover design n=4 horses were administered either 8L of a balanced hypotonic oral electrolyte solution (**PNW-8**), or water alone (**H_2O-8**), 60 min before they exercised on a treadmill at 50% of peak VO_2 for 45 minutes. Variables were measured using a NovaStatProfile 5+ bloodgas analyzer, and calculated using AcidBasicsII software.

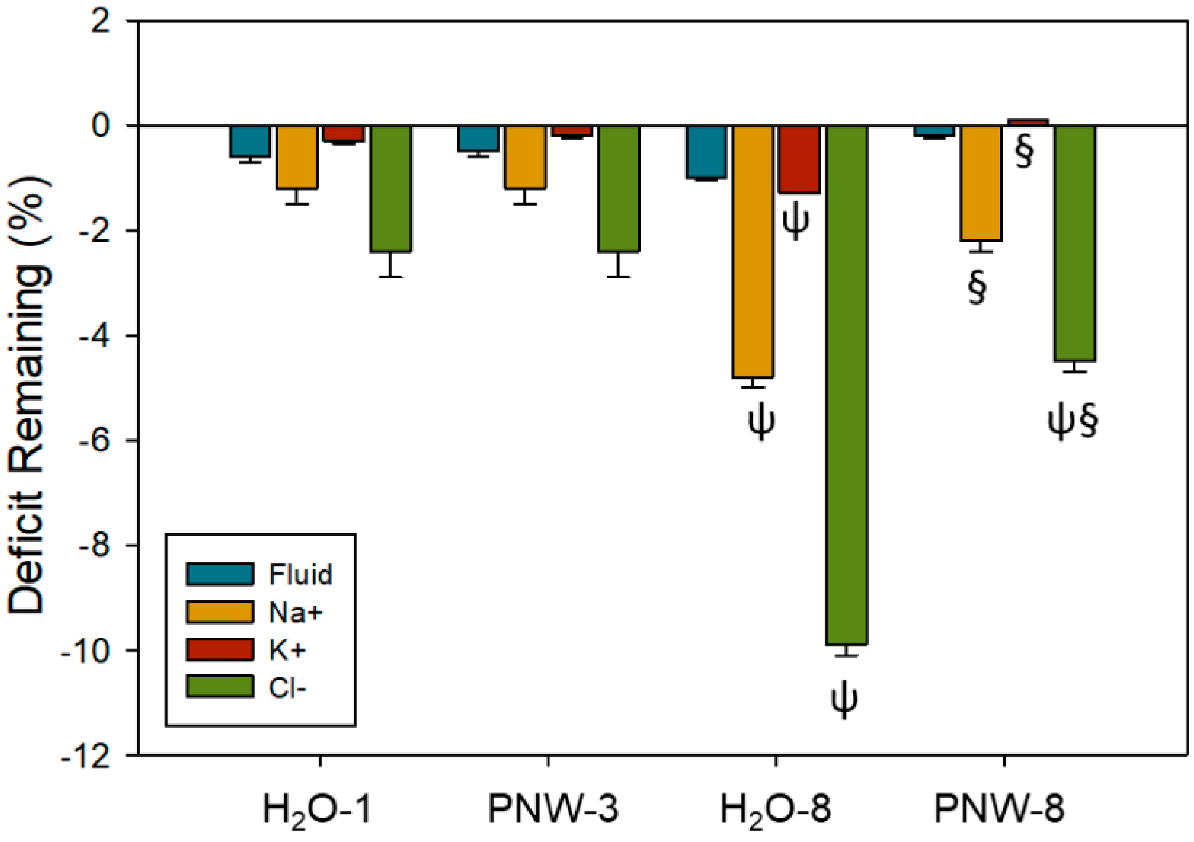

Figure S3: Total body deficit of fluid and electrolytes at the end of the sampling period. Deficit remaining was calculated as the difference between the cumulative sweat losses and the fluid and ions added by pre-loading, and expressed as a percentage of total body stores pre-exercise. Values are Mean ± SEM for n=4 horses.

Ψ, § indicate significantly different from the H_2O-1 and H_2O-8 trials, respectively.
